# Stigma‐Related Experiences and Their Associations With Sociodemographic and Clinical Characteristics Among Breast Cancer Survivors in a Tertiary Care Hospital, Muzaffarabad: A Cross‐Sectional Study

**DOI:** 10.1002/hsr2.73197

**Published:** 2026-09-03

**Authors:** Nimra Gillani, Saman Waqar, Mudassir Kamal, Muhammad Iltaf, Amanullah Khan, Muhammad Hassaan Javaid, Hasibullah Aminpoor

**Affiliations:** ^1^ National Institue of Health Islamabad Pakistan; ^2^ Al‐ Shifa Trust Rawalpindi Pakistan; ^3^ Armed Forces Institute of Cardiology Rawalpindi Pakistan; ^4^ Ayub Teaching Hospital Abbottabad Pakistan; ^5^ Ayub Medical College Abbottabad Pakistan; ^6^ Shifa College of Medicine Islamabad Pakistan; ^7^ Faculty of Medicine Kabul University of Medical Sciences “Abu Ali Ibn Sina” Kabul Afghanistan; ^8^ Resident Physician, Department of Internal Medicine Rabia Balkhi National Complex Hospital Kabul Afghanistan

**Keywords:** breast cancer survivors, financial hardship, psychological impact, social rejection, stigma

## Abstract

**Background:**

Breast cancer is the most commonly diagnosed cancer among women worldwide and remains a major cause of cancer‐related morbidity and mortality. Cancer‐related stigma may adversely affect social relationships, psychological well‐being, and financial security. Stigma affects the quality of life, treatment adherence, and mental health. The present study examined the associations between participant characteristics and stigma among breast cancer survivors in Muzaffarabad.

**Methods:**

A total of 124 women aged ≥ 18 years with stage I–III breast cancer were recruited through consecutive non‐probability sampling, of whom 101 with complete responses were included in the final analysis. The data were collected using a questionnaire adapted from Jin et al. (2021) and included questions on socio‐demographics and factors associated with stigma, which were evaluated using components of the social impact scale, such as social rejection, financial burden, and psychological impact. Data were analyzed using Jamovi version 2.6.44.0. Associations between continuous stigma domain scores and participants' sociodemographic and clinical characteristics were assessed using Mann–Whitney U and Kruskal–Wallis tests. Statistical significance was set at *p* < 0.05.

**Results:**

The majority of respondents were aged 45–59 years (59.4%), married (88.0%), and housewives (78.2%). Mean domain scores were 21.1 ± 3.9 for social rejection (possible range 7–28), 6.1 ± 0.5 for financial hardship (2–8), and 36.4 ± 4.5 for psychological impact (12–48). Because the domains contain different numbers of items, these scores should not be interpreted as direct comparisons of stigma burden. Financial hardship scores differed across disease stages. However, no clear directional trend was observed, and the small number of participants in Stage I limits the interpretation of subgroup differences. Therefore, this finding should be considered exploratory.

**Conclusion:**

Breast cancer survivors experienced substantial stigma‐related experiences across social, financial, and psychological domains. Although most sociodemographic and clinical characteristics were not significantly associated with stigma scores, financial hardship varied significantly across disease stages. However, because the Stage I subgroup was very small, these findings should be interpreted cautiously, and the observed differences do not indicate a consistent increase in financial hardship with advancing disease stage.

## 1 Introduction

Cancer is a global concern, with breast cancer being especially prevalent among women. It is defined as uncontrolled cell growth. Breast cancer begins in the milk ducts or lobules and can spread to other tissues in the body. Metastasis may occur in other organs and cause death. Breast cancer is the most prevalent form of cancer in women in 157 out of 185 countries in 2022 [1].

As per 2020 data, breast cancer became the most common cancer type globally, showing a sharp increase in its prevalence. As per the statistics of Pink Ribbon Oncologist in 2022, Pakistan has the highest prevalence rate of breast cancer cases, that is, 22.9%, and it constitutes a major part of the burden of cancers. Specifically, Pakistan carries a high burden of breast cancer; one in ten women is likely to develop breast cancer in her life. While higher incidence is found in Western European countries, higher mortality rates were reported in less developed countries such as Pakistan due to late detection [2].

However, besides the threat to physical well‐being, breast cancer patients may suffer from discrimination due to the prevailing stigmatization, caused by false information, changes in self‐image, and cultural beliefs. Cultural beliefs, including the belief that cancer is a contagious disease, as well as that it is caused by personality defects, contribute to the social isolation of patients and their families [3].

Discrimination takes various forms, ranging from social discrimination to financial strain and psychological impact, thus hampering rehabilitation and quality of life. While there have been many studies on stigma among cancer survivors around the globe, there is limited literature about associated factors in the case of Pakistan. This knowledge is essential for developing effective intervention strategies to help patients cope psychologically and eliminate misconceptions.

The objectives of this study were to assess stigma across three domains and examine its associations with sociodemographic and clinical characteristics. This research would hopefully help develop culturally appropriate interventions to assist the victims and combat the issues associated with stigma in Pakistan.

## 2 Methods

### 2.1 Study Design

This study used a hospital‐based cross‐sectional analysis designed to evaluate stigmatization levels and investigate the relationship between stigmatization levels and sociodemographic and clinical factors among breast cancer survivors. Given the cross‐sectional study design, causal relationships cannot be established. Therefore, the findings are presented as associations between stigma domain scores and participant characteristics rather than as predictors or determinants of stigma. Because the analyses examined multiple associations across several participant characteristics and three stigma domains, the analyses were considered exploratory. No formal adjustment for multiple comparisons was applied; therefore, *p* values should be interpreted cautiously, and the isolated statistically significant finding should be regarded as hypothesis‐generating rather than confirmatory.

### 2.2 Study Setting

This study was conducted at a tertiary care hospital in Muzaffarabad, Azad Kashmir, over 6 months from September 2023 to February 2024.

### 2.3 Study Population

The participants of this study included female breast cancer survivors who came for their follow‐up either while being treated or post‐treatment.

### 2.4 Eligibility Criteria

#### 2.4.1 Inclusion Criteria

Female breast cancer survivors aged 18 years or older with a diagnosis of histopathologically proven breast cancer (I–III stages) who had received treatment for at least 6 months and who had no documented recurrence at the time of data collection were eligible.

#### 2.4.2 Exclusion Criteria

Males, pregnant females, non‐compliant subjects, patients with benign breast conditions, and those with recurrence after 1 month post‐treatment.

### 2.5 Sample Size

Sample size was calculated using OpenEpi version 3, assuming an expected prevalence of 7.8%, which was obtained from Jin et al. [3], a 5% absolute margin of error, and a 95% confidence level. The calculated minimum sample size was 111 participants. Allowing for 10% potential non‐response or incomplete responses, the target sample size was increased to 124 participants. A total of 124 participants were recruited; however, 23 participants had incomplete responses and were excluded from the final analysis. Thus, 101 participants were included in the final analysis.

### 2.6 Sampling Strategy

Non‐probability consecutive sampling was used.

### 2.7 Data Collection Instrument

Data were collected using an adapted version of the Social Impact Scale (SIS) based on the three‐domain structure described by Jin et al. [3]. The tool was translated into the local language using forward–backward translation by bilingual experts to ensure semantic equivalence. The questionnaire comprised 31 items, including 10 sociodemographic questions and 21 stigma‐related items adapted from the Social Impact Scale (SIS). The stigma items were organized into three domains: Social Rejection (7 items), Financial Hardship (2 items), and Psychological Impact (12 items). Each item was scored on a 4‐point Likert scale:

1 = Strongly disagree

2 = Disagree

3 = Agree

4 = Strongly agree

Higher scores indicated higher perceived stigma.

### 2.8 Scoring and Operational Definition of Stigma

#### 2.8.1 Operational Definition and Scoring of Stigma

In this study, stigma was operationally defined using the Social Impact Scale (SIS). The SIS measures perceived stigma across three domains: social rejection, financial hardship, and psychological impact. Each item was scored on a Likert scale, and domain scores were calculated by summing responses to items within each domain. The possible score ranges were 7–28 for the Social Rejection domain, 2–8 for the Financial Hardship domain, and 12–48 for the Psychological Impact domain. Higher domain scores indicated greater perceived stigma within the respective domain. Because the three domains contain different numbers of items and different possible score ranges, raw summed scores were not interpreted as direct measures of the relative magnitude of stigma across domains.

For analysis, stigma was assessed using continuous domain‐specific scores for social rejection, financial hardship, and psychological impact, derived from the Social Impact Scale.

### 2.9 Data Collection Procedure

Ethical clearance was obtained from the Al‐Shifa School of Public Health (ethical approval code: MSPH‐IRB/16‐06). Data collection involved physical visits to hospitals as well as the use of self‐administered questionnaires. Individual participants were selected one at a time, and the study's aim was clearly communicated before data collection.

### 2.10 Pilot Testing

The pilot study involved 10% of the subjects in the study. No significant revisions had to be made after conducting the pilot study, and data collected during the pilot study were not included in the analysis of data.

### 2.11 Reliability

Internal consistency of the adapted Social Impact Scale was assessed using Cronbach's alpha. The overall adapted social impact scale demonstrated excellent internal consistency (Cronbach's *α* = 0.91). Domain‐specific Cronbach's alpha coefficients were *α* = 0.91 for Social Rejection, *α* = 0.60 for Financial Hardship, and *α* = 0.92 for Psychological Impact. The lower alpha observed for the Financial Hardship domain is interpreted with caution because the subscale comprises only two items, and Cronbach's alpha is known to underestimate reliability for very short scales. No additional psychometric analyses, such as construct validity, factor analysis, or floor and ceiling effects, were performed.

### 2.12 Data Analysis

Analysis was performed with Jamovi version 2.6.44.0. Descriptive statistics included frequencies and percentages for categorical variables. Because stigma domain scores were analyzed using non‐parametric methods, medians and interquartile ranges (IQRs) were used as the primary descriptive statistics for continuous domain scores. Means and standard deviations were also reported to facilitate comparison with previous studies. Domain scores for social rejection, financial hardship, and psychological impact were calculated by summing item responses within each domain of the Social Impact Scale (SIS). These domain scores were analyzed as continuous variables. Because the distributions of the domain scores did not satisfy the assumptions required for parametric comparisons and several subgroups were small, non‐parametric tests were used. Mann–Whitney U tests were used for comparisons involving two groups, whereas Kruskal–Wallis tests were used for comparisons involving more than two groups. Statistical significance was set at *p* < 0.05. It should be noted that due to its cross‐sectional nature, findings represent associations rather than predictors. Effect sizes were reported as epsilon‐squared (*ε* ^2^) for Kruskal–Wallis tests and rank‐biserial correlation (rrb) for Mann–Whitney U tests.

### 2.13 Ethical Considerations

This research was conducted in accordance with the principles of the Declaration of Helsinki. Approval by the IRB and consent forms from all participants were obtained. The privacy and confidentiality of the data were ensured.

The respondents were given the freedom to opt out of participating in the study at any point. This study sought to identify sociodemographic and clinical characteristics associated with stigma among breast cancer survivors.

## 3 Results

### 3.1 Sociodemographic and Clinical Characteristics

Of the 124 participants recruited for the study, 23 were excluded because of incomplete responses. Thus, 101 participants were included in the final analysis. The largest proportion of participants was aged 45–59 years (59.4%), followed by those aged ≥ 60 years (31.7%) and 31–44 years (8.9%). No participants were aged 18–30 years. Most of the subjects had matric education (38.6%), and they were married (88%). Economically, most of them earned monthly from 30,000 to 50,000 PKR (45.5%), and 78.2% were housewives.

With respect to clinical characteristics, most patients belonged to stage III disease (64.4%) and had undergone mastectomy (56.4%). Chemotherapy was received by 77.2% of participants, whereas 29.7% underwent radiotherapy (Table 1).

**Table 1:** Sociodemographic and clinical characteristics of breast cancer survivors (*N* = 101).

Characteristics	Number (%)
Age (years)	
31–44	9 (8.9)
45–59	60 (59.4)
≥ 60	32 (31.7)
Education level	
Under matric	32 (31.7)
Matric	39 (38.6)
Intermediate	24 (23.8)
Bachelor's or higher	6 (5.9)
Marital status	
Married	89 (88.1)
Other*	12 (11.9)
Monthly income	
< 30,000 PKR	24 (23.8)
30,000–50,000 PKR	46 (45.5)
> 50,000 PKR	31 (30.7)
Employment status	
Employed	22 (21.8)
Housewife	79 (78.2)
Disease stage	
Stage I	3 (3.0)
Stage II	33 (32.7)
Stage III	65 (64.4)
Survival time (months)	
< 12	32 (31.7)
12–36	50 (49.5)
37–60	15 (14.9)
61–120	3 (3.0)
> 120	1 (1.0)
Surgery type	
Mastectomy	57 (56.4)
Breast conservation/reconstruction	44 (43.6)
Radiotherapy	
Yes	30 (29.7)
No	71 (70.3)
Chemotherapy	
Yes	78 (77.2)
No	23 (22.8)

*Note:* Others* include unmarried, divorced, and widowed participants.

### 3.2 Stigma Domain Scores

Stigma was assessed across three domains: Social Rejection, Financial Hardship, and Psychological Impact. Median (interquartile range [IQR]) scores were 23 [4] for Social Rejection, 6 (0) for Financial Hardship, and 37 [5] for Psychological Impact. Corresponding mean ± standard deviation values were 21.1 ± 3.9, 6.1 ± 0.5, and 36.4 ± 4.5, respectively (Table 2). The possible score ranges were 7–28, 2–8, and 12–48, respectively. Because the three domains comprise different numbers of items and different possible score ranges, the raw summed scores should not be interpreted as direct comparisons of stigma burden across domains (Table 2).

**Table 2:** Distribution of stigma domain scores among participants.

Domain	Median (IQR)	Mean ± SD	Possible score range	Observed range
Social rejection	23 (5)	21.1 ± 3.9	7–28	12–28
Financial hardship	6 (0)	6.1 ± 0.5	2–8	5–8
Psychological impact	37 (2)	36.4 ± 4.5	12–48	24–48

*Note:* The adapted Social Impact Scale demonstrated excellent overall internal consistency (Cronbach's *α* = 0.91). Domain‐specific Cronbach's alpha coefficients were 0.91 for Social Rejection, 0.60 for Financial Hardship, and 0.92 for Psychological Impact.

### 3.3 Item‐Wise Stigma Experiences

Many participants indicated stigma experiences in social, economic, and psychological aspects. Over eight out of ten experienced a lack of respect (82.2%) and felt rejected by family members and friends (76.2%). Likewise, 79.2% indicated that people think of their illness as contagious, and 82.2% experienced exclusion due to stigma.

Economic impact was an important aspect among participants since all participants indicated at least one experience associated with financial strain. Further, 96.0% of participants said that financial strain impacted their relationships.

Other psychological consequences were also indicated by participants. These included feeling blamed (82.2%), lonely (82.2%), problems in communicating openly (82.2%), and being a burden on family members (100%). Other frequently mentioned aspects were feeling inadequate after the operation and requiring reassurance (Table 3 and Supporting Information S1: Table S1).

**Table 3:** Item‐wise prevalence of stigma‐related experiences among participants (*N* = 101) (agree + strongly agree combined).

Item (shortened)	Agree/strongly agree *n* (%)
Felt treated with less respect	83 (82.2)
Felt rejected by family/friends	77 (76.2)
Felt less competent in the eyes of others	83 (82.2)
Others thought illness was contagious	80 (79.2)
Felt avoided due to illness	80 (79.2)
Experienced embarrassment due to illness	83 (82.2)
Others felt awkward around you	83 (82.2)
Experienced financial hardship	93 (92.1)
Financial hardship affected relationships	97 (96.0)
Felt blamed for illness	83 (82.2)
Difficulty being open about illness	83 (82.2)
Fear of disclosure of illness	83 (82.2)
Felt lonely due to illness	83 (82.2)
Needed more reassurance	90 (89.1)
Felt unequal in relationships	94 (93.1)
Felt like a burden on the family	101 (100.0)
Appearance affected relationships	97 (96.0)
Felt imperfect after surgery	101 (100.0)
Fear of intimate physical contact	97 (96.0)
Difficulty adapting to limitations	101 (100.0)
Felt useless due to illness	83 (82.2)

*Note:* Full item‐wise responses to the stigma scale using the 4‐point Likert format are provided in Supporting Information S1: Table S1.

### 3.4 Association Between Stigma Domains and Sociodemographic Characteristics

Associations between stigma domain scores and participant characteristics were examined using Mann–Whitney U and Kruskal–Wallis tests. Most sociodemographic and clinical variables were not significantly associated with social rejection, financial hardship, or psychological impact scores. Disease stage was significantly associated with financial hardship scores (Kruskal–Wallis *H* = 10.884, df = 2, *p* = 0.004, *ε* ^2 ^= 0.109). Post‐hoc Dwass–Steel–Critchlow–Fligner comparisons showed significant differences between Stage I and Stage II (*p *= 0.017) and between Stage I and Stage III (*p *= 0.003), whereas the difference between Stage II and Stage III was not statistically significant (*p *= 0.904). Because the Stage I subgroup comprised only three participants, this finding should be interpreted cautiously and should not be interpreted as evidence of a progressive increase in financial hardship with advancing disease stage. Likewise, age, education, income, survival time, marital status, employment status, radiotherapy, chemotherapy, and surgery type were not significantly associated with stigma domain scores (Table 4, Supporting Information S2: Table S2).

**Table 4:** Association of participants' sociodemographic and clinical characteristics and stigma domain scores.

Variable	Social rejection	Financial hardship	Psychological impact
Age	*χ* ^2^ (2) = 0.03, *p* = 0.988, *ε* ^2 ^< 0.001	*χ* ^2^ (2) = 0.20, *p* = 0.907, ε^2 ^= 0.002	*χ* ^2^ (2) = 3.39, *p* = 0.184, *ε* ^2 ^= 0.034
Education	*χ* ^2^ (3) = 4.99, *p* = 0.172, *ε* ^2 ^= 0.020	*χ* ^2^ (3) = 2.97, *p* = 0.397, *ε* ^2 ^= 0.000	*χ* ^2^(3) = 0.04, *p* = 0.998, *ε* ^2 ^< 0.001
Monthly income	*χ* ^2^ (2) = 0.03, *p* = 0.983, *ε* ^2 ^< 0.001	*χ* ^2^ (2) = 2.12, *p* = 0.347, *ε* ^2 ^= 0.021	*χ* ^2^ (2) = 0.45, *p* = 0.800, *ε* ^2 ^= 0.004
Disease stage	*χ* ^2^ (2) = 0.99, *p* = 0.610, *ε* ^2 ^= 0.010	*χ* ^2^ (2) = 10.88, *p* = 0.004, *ε* ^2^ = 0.109	*χ* ^2^ (2) = 1.18, *p* = 0.556, *ε* ^2 ^= 0.012
Survival time	*χ* ^2^ (4) = 4.49, *p* = 0.344, *ε* ^2 ^= 0.045	*χ* ^2^ (4) = 6.48, *p* = 0.166, *ε* ^2 ^= 0.065	*χ* ^2^ (4) = 8.22, *p* = 0.084, *ε* ^2 ^= 0.082
Employment status	*U* = 225, *p* = 0.385, r(rb) = −0.211	*U* = 246, *p* = 0.325, r(rb) = −0.137	*U* = 276, *p* = 0.899, r(rb) = −0.032
Marital status	*U* = 506, *p* = 0.765, r(rb) = −0.053	*U* = 511, *p* = 0.674, r(rb) = −0.043	*U* = 479, *p* = 0.549, r(rb) = −0.104
Radiotherapy	*U* = 877, *p* = 0.157, r(rb) = −0.177	*U* = 1056, *p* = 0.910, r(rb) = 0.008	*U* = 926, *p* = 0.286, r(rb) = −0.131
Chemotherapy	*U* = 747, *p* = 0.219, r(rb) = 0.167	*U* = 892, *p* = 0.948, r(rb) = 0.006	*U* = 765, *p* = 0.269, r(rb) = 0.147
Surgery type	*U* = 1144, *p* = 0.444, r(rb) = −0.088	*U* = 1239, *p* = 0.860, r(rb) = 0.012	*U* = 1196, *p* = 0.683, r(rb) = −0.046

*Note:* The association had an *ε* ^2^ of 0.109, indicating that disease stage accounted for approximately 10.9% of the variability represented by the Kruskal–Wallis effect‐size estimate. Because interpretation thresholds vary across conventions and the Stage I subgroup was very small, the effect size is reported without assigning a qualitative label. Post‐hoc Dwass–Steel–Critchlow–Fligner pairwise comparisons were performed only for statistically significant Kruskal–Wallis tests.

Abbreviations: *χ* ^2^, Kruskal–Wallis test statistic; U, Mann–Whitney U statistic; *ε* ^2^, epsilon‐squared effect size; r_(_rb_)_, rank‐biserial correlation.

## 4 Discussion

The study participants reported stigma experiences across social rejection, financial hardship, and psychological impact domains. There was a presence of severe psychological stigma, which is evidenced by the high score in the psychological domain. It is worth noting that as there are different numbers of items in each domain, direct comparison of the domain scores is not feasible.

While many participants reported a high level of social rejection, there were no statistically significant correlations of the social rejection scores with the socioeconomic/clinical characteristics examined in this study. There could be a possibility that social rejection is a universal experience of breast cancer survivors independent of their age, income, employment, treatments, and disease characteristics. Previously, similar conclusions have been reached before where the stigma and altered social interaction were found to be an issue among breast cancer survivors regardless of demographic factors and populations involved [3, 4]. The above results underscore the significance of community‐based awareness and patient‐oriented programs aimed at reducing cancer stigma and social isolation.

The financial hardship domain was the most common in the study participants. There was a significant relationship between the disease stage and financial hardship scores (*H* = 10.884, *p* = 0.004, *ε* ^2 ^= 0.109). Post hoc analyses revealed significant differences in a small subgroup of the Stage I group, while no difference was identified between Stage II and Stage III. Therefore, the relationship does not reflect the progressive increase of financial burden associated with advancing disease stages. As only three participants belonged to the Stage I group, the above finding can be treated as an exploratory one and should be interpreted cautiously. The effect size was presented without being labeled as small or moderate due to variations in *ε* ^2^ interpretation conventions, and the sparse Stage I subgroup may affect the stability of the estimate.

There were high psychological impact scores amongst the participants, which signified high experiences of psychological distress; however, direct comparisons could not be made since raw domain scores differed due to differences in the number of items in each domain. There was no relation between psychological impact scores and sociodemographic/clinical characteristics of the participants; nonetheless, the generally high psychological distress burden indicates that there is emotional distress among BC patients. Fears of cancer relapse, concerns over body image, concerns about the future, and disruption of social/familial roles account for most psychological distress in cancer patients [6, 7]. These findings emphasize the need for integrating psychosocial evaluation, counseling, and psychological care into routine oncology practice.

There were high stigmatization experiences among the BC survivors in the social, economic, and psychological domains. Even though very few associations between stigmatization domain scores and sociodemographic/clinical characteristics were reported, disease stage was associated with financial strain scores. However, due to the small size of the subgroup with stage I disease and the lack of a trend, this finding must be considered cautiously as exploratory. Since the study was cross‐sectional, the associations observed do not indicate predictors or determinants of stigma experiences.

### 4.1 Limitations

There are some limitations of this study. Firstly, the cross‐sectional nature of the research precluded the possibility of causal inference and establishment of the temporal relationship between stigma and the factors related to it. Secondly, the utilization of one‐center samples might reduce the external validity of the results. Thirdly, there might be low statistical power due to the relatively small sample size used to assess the associations. The study had a relatively small number of participants in the final analysis sample, with 101 out of 124 recruited subjects giving their complete responses. Consequently, the exclusion of participants with incomplete answers might lead to the selection bias. Fourthly, stigma was assessed by self‐reporting, which might increase the susceptibility of the results to the social desirability bias. The number of participants in some subgroups was quite small and could influence the precision and stability of the statistical estimates. Thus, the results should be interpreted cautiously and confirmed using multicentre large‐sample studies. Although the adapted Social Impact Scale showed excellent internal consistency (Cronbach's *α* = 0.90), the reliability of the individual domains of the scale differed. The Social Rejection (*α* = 0.91) and Psychological Impact (*α* = 0.92) domains revealed excellent internal consistency, while the domain Financial Hardship revealed lower reliability (*α* = 0.60). This relatively low coefficient should be interpreted cautiously since this particular domain included only two items and, as is known, Cronbach's alpha underestimated the reliability in the case of a small number of items. In addition, other psychometric properties, such as construct validity, factor structure, and presence of the floor/ceiling effects, were not established. The study may have been underpowered to detect small associations. Future studies should undertake comprehensive psychometric validation of the adapted instrument in the Pakistani population.

## 5 Conclusion

This study demonstrated that breast cancer survivors attending a tertiary care hospital in Muzaffarabad reported stigma‐related experiences across social, financial, and psychological domains. Most sociodemographic and clinical characteristics were not significantly associated with stigma domain scores. Financial hardship scores differed across disease stages; however, the pattern did not demonstrate a consistent increase with advancing stage, and the small Stage I subgroup limits interpretation. This finding should therefore be considered exploratory. Our cross‐sectional study design, however, suggests that these relationships should be interpreted as associations, not causality or prediction. Multiple subgroup comparisons were performed without adjustment; therefore, isolated statistically significant findings should be interpreted cautiously. Large multicentre studies will be necessary to better elucidate determinants of stigma among breast cancer survivors and guide intervention strategies.

## 6 Recommendations

1. Integrate psychosocial counseling, mental health support, and routine stigma screening into standard breast cancer survivorship care to identify and address stigma‐related concerns early.
2. Implement community awareness campaigns and healthcare provider training to reduce misconceptions, discrimination, and stigma while promoting patient‐centered communication and care.
3. Strengthen financial assistance programs and establish breast cancer survivor support groups to reduce treatment‐related financial hardship and enhance social support and coping.
4. Conduct larger multicenter studies using robust multivariable analytical approaches to better understand factors associated with stigma among breast cancer survivors.
5. Validate culturally appropriate stigma assessment tools and undertake qualitative research to explore the sociocultural beliefs and lived experiences that contribute to breast cancer‐related stigma in Pakistan.

## Author Contributions

Nimra Gillani was involved in conceptualization, data curation, supervision, and project administration. Saman Waqar and Mudassir Kamal critically evaluated the literature on project administration. Muhammad Iltaf and Amanullah Khan did the final review and analysis. Muhammad Hassaan Javaid and Hasibullah Aminpoor visualized and drafted the manuscript. All authors read and approved the final manuscript.

## Funding

The authors have nothing to report.

## Ethics Statement

An IRB approval was obtained from the ethical committee of Al Shifa School of Public Health with reference number MSPH‐IRB/16‐06 after the synopsis presentation. An informed consent form is attached in the annexure that was signed by every participant before data collection. The information collected from the participants was only to be used for the purpose of research. All the information and data were kept strictly confidential. There was no risk in this research.

## Consent

Consent was taken from every participant before data collection.

## Conflicts of Interest

The authors declare no conflicts of interest.

## Transparency Statement

The corresponding author, Hasibullah Aminpoor, affirms that this manuscript is an honest, accurate, and transparent account of the study being reported; that no important aspects of the study have been omitted; and that any discrepancies from the study as planned (and, if relevant, registered) have been explained.

## Supporting information


Supporting File 1

Supporting File 2

## Data Availability

Data are available upon reasonable request from the authors.
