# Supplementary material for: Stigma‐Related Experiences and Their Associations With Sociodemographic and Clinical Characteristics Among Breast Cancer Survivors in a Tertiary Care Hospital, Muzaffarabad: A Cross‐Sectional Study
Source: Health Sci Rep. 2026 Sep 3;9(9):e73197. doi: 10.1002/hsr2.73197 (PMC13542179; doi:10.1002/hsr2.73197)
Supplement: Supplementary file 1 [file HSR2-9-e73197-s001.docx]

**Supplementary Table S2. Median (Interquartile Range) Scores for Social Rejection, Financial Hardship, and Psychological Impact Domains According to Participant Characteristics**

| **Characteristic** | **Category** | **Social Rejection Median (IQR)** | **Financial Hardship Median (IQR)** | **Psychological Impact Median (IQR)** |
| --- | --- | --- | --- | --- |
| **Age (years)** | 31–44 | 23 (8.0) | 6 (0.0) | 37 (6.0) |
|  | 45–59 | 23 (5.0) | 6 (0.0) | 37 (2.0) |
|  | ≥60 | 22 (2.0) | 6 (0.0) | 37 (1.0) |
| **Education** | Under matric | 23 (4.5) | 6 (0.0) | 37 (4.0) |
|  | Matric | 23 (2.0) | 6 (0.0) | 37 (2.0) |
|  | Intermediate | 22 (5.0) | 6 (0.0) | 37 (0.25) |
|  | Bachelor's degree OR higher | 18 (3.0) | 6 (0.0) | 37 (0.0) |
| **Monthly income (PKR)** | <30,000 | 22 (2.0) | 6 (0.0) | 37 (2.0) |
|  | 30,000–50,000 | 22.5 (5.75) | 6 (0.0) | 37 (2.0) |
|  | >50,000 | 23 (4.25) | 6 (0.0) | 37 (1.5) |
| **Disease stage** | Stage I | 25 (4.5) | 7 (1.0) | 41 (7.0) |
|  | Stage II | 22 (5.0) | 6 (0.0) | 37 (2.0) |
|  | Stage III | 23 (2.0) | 6 (0.0) | 37 (2.0) |
| **Survival time** | <12 months | 23 (5.5) | 6 (0.0) | 37 (2.25) |
|  | 12–36 months | 22.5 (5.0) | 6 (0.0) | 37 (1.75) |
|  | 37–60 months | 22 (2.5) | 6 (0.0) | 37 (1.5) |
|  | 61–120 months | 26 (3.0) | 6 (0.5) | 41 (5.0) |
|  | >120 months (n=1) | 18 (0.0) | 6 (0.0) | 37 (0.0) |
| **Employment status** | Full-time employment | 23.5 (7.0) | 6 (0.0) | 37 (7.5) |
|  | Housewife | 23 (5.0) | 6 (0.0) | 37 (2.0) |
| **Marital status** | Married | 23 (5.0) | 6 (0.0) | 37 (2.0) |
|  | Unmarried/divorced/widowed | 22.5 (7.75) | 6 (0.0) | 37 (7.5) |
| **Radiotherapy** | Yes | 23 (1.0) | 6 (0.0) | 37 (1.0) |
|  | No | 22 (5.0) | 6 (0.0) | 37 (2.0) |
| **Chemotherapy** | Yes | 22 (5.0) | 6 (0.0) | 37 (2.0) |
|  | No | 23 (1.0) | 6 (0.0) | 37 (1.0) |
| **Surgery type** | Mastectomy | 23 (6.0) | 6 (0.0) | 37 (2.0) |
|  | Breast-conserving surgery/reconstruction | 22 (5.0) | 6 (0.0) | 37 (2.0) |
