# Supplementary material for: Stigma‐Related Experiences and Their Associations With Sociodemographic and Clinical Characteristics Among Breast Cancer Survivors in a Tertiary Care Hospital, Muzaffarabad: A Cross‐Sectional Study
Source: Health Sci Rep. 2026 Sep 3;9(9):e73197. doi: 10.1002/hsr2.73197 (PMC13542179; doi:10.1002/hsr2.73197)
Supplement: Supplementary file 2 [file HSR2-9-e73197-s002.docx]

**Table S1. Full distribution of responses to stigma scale items**

| **Domain** | **Item (shortened)** | **Strongly Disagree n (%)** | **Disagree n (%)** | **Agree n (%)** | **Strongly Agree n (%)** |
| --- | --- | --- | --- | --- | --- |
| Social rejection | Felt less respected | 0 (0.0) | 18 (17.8) | 45 (44.6) | 38 (37.6) |
| Social rejection | Rejected by family/friends | 0 (0.0) | 24 (23.8) | 55 (54.5) | 22 (21.8) |
| Social rejection | Viewed as less competent | 0 (0.0) | 18 (17.8) | 75 (74.3) | 8 (7.9) |
| Social rejection | Believed illness is contagious | 4 (4.0) | 17 (16.8) | 58 (57.4) | 22 (21.8) |
| Social rejection | Avoided by others | 4 (4.0) | 17 (16.8) | 52 (51.5) | 28 (27.7) |
| Social rejection | Embarrassed due to illness | 4 (4.0) | 14 (13.9) | 59 (58.4) | 24 (23.8) |
| Social rejection | Others felt awkward around you | 0 (0.0) | 18 (17.8) | 61 (60.4) | 22 (21.8) |
| Financial hardship | Financial hardship experienced | 0 (0.0) | 0 (0.0) | 93 (92.1) | 8 (7.9) |
| Financial hardship | Affected relationships | 0 (0.0) | 4 (4.0) | 94 (93.1) | 3 (3.0) |
| Psychological impact | Felt blamed for illness | 0 (0.0) | 18 (17.8) | 68 (67.3) | 15 (14.9) |
| Psychological impact | Difficulty being open | 0 (0.0) | 18 (17.8) | 72 (71.3) | 11 (10.9) |
| Psychological impact | Fear of disclosure | 4 (4.0) | 14 (13.9) | 64 (63.4) | 19 (18.8) |
| Psychological impact | Felt lonely | 0 (0.0) | 18 (17.8) | 68 (67.3) | 15 (14.9) |
| Psychological impact | Need reassurance | 4 (4.0) | 7 (6.9) | 85 (84.2) | 5 (5.0) |
| Psychological impact | Felt unequal in relationships | 0 (0.0) | 7 (6.9) | 86 (85.1) | 8 (7.9) |
| Psychological impact | Felt burden on family | 0 (0.0) | 0 (0.0) | 83 (82.2) | 18 (17.8) |
| Psychological impact | Appearance affected relationships | 4 (4.0) | 0 (0.0) | 93 (92.1) | 4 (3.9) |
| Psychological impact | Felt imperfect after surgery | 0 (0.0) | 0 (0.0) | 86 (85.1) | 15 (14.9) |
| Psychological impact | Fear of physical intimacy | 0 (0.0) | 4 (4.0) | 89 (88.1) | 8 (7.9) |
| Psychological impact | Difficulty adapting to illness | 0 (0.0) | 0 (0.0) | 60 (59.4) | 41 (40.6) |
| Psychological impact | Felt useless due to illness | 4 (4.0) | 14 (13.9) | 71 (70.3) | 12 (11.9) |
